# Advances in Wearable Photoplethysmography Applications in Health Monitoring

**DOI:** 10.3390/s23167064

**Published:** 2023-08-10

**Authors:** Mimma Nardelli, Raquel Bailón

**Affiliations:** 1 Bioengineering and Robotics Research Centre “E. Piaggio” and Dipartimento di Ingegneria dell’Informazione, University of Pisa, Largo Lucio Lazzarino 1, 56122 Pisa, Italy; 2 BSICoS Group, Aragón Institute of Engineering Research (I3A), IISAragon, University of Zaragoza, 50015 Zaragoza, Spain; rbailon@unizar.es; 3 Biomedical Research Networking Center in Bioengineering, Biomaterials and Nanomedicine (CIBER-BBN), 28029 Madrid, Spain

## 1. Introduction

In the last few years, interest in wearable technology for physiological signal monitoring is rapidly growing, especially during and after the COVID-19 pandemic [1,2,3]. Specifically, considering that heart disease is the leading cause of death globally, continuous monitoring of cardiovascular dynamics has crucial relevance to improving prevention and diagnosis. Photoplethysmography (PPG) is a popular, non-invasive, and low-cost optical technique that can provide useful information about the cardiovascular system, aiming to reveal autonomic dysfunctions and peripheral vascular diseases during daily life. In fact, due to its simplicity and versatility, this technology can be used to develop wearable and wireless devices for out-of-hospital monitoring of both healthy and pathological subjects.

Even if technology has successfully increased the comfort of PPG sensors, in terms of wearability, dimensions and battery life, scientific research is still working on several issues, e.g., poor sensor contact, which leads to acquiring signals corrupted by noise and motion artifacts, especially during physical activity [4]. In this context, there are still many challenges related to PPG wearable device design and signal processing techniques to derive robust indices. Furthermore, recent studies have shed light on the possibility of extracting a good surrogate of PPG signal from face RGB video processing, opening the door to not only wireless but also contactless monitoring. For this reasons, the investigation of reliable PPG-derived parameters, including rhythm and morphology features, but also heart rate variability descriptors, is growing in interest, comprising novel signal processing methodologies for artifact removal and feature extraction.

This Special Issue focused on original research papers dealing with hardware and software advances in the development of robust and reliable biomarkers for the non-invasive monitoring of cardiovascular dynamics based on PPG signal acquisition. Topics of interest for PPG signal applications included clinical pathologies, biometry, sleep and sport monitoring.

## 2. Contributions

In the current Special Issue, the optimization of hardware and software systems for a reliable PPG signal acquisition and pre-processing was one of the main topics. The influence of the light source wavelength of the PPG sensor on the accuracy of blood pressure estimation was investigated by Toda et al. [5], who proposed a new system based on four light-emitting diodes (near-infrared (NIR), red, green, and blue) acquiring multi-wavelength PPG signals from the index finger. NIR was found to be the most accurate wavelength for the estimation of systolic blood pressure from PPG signals, and blue was the best performing when they extracted diastolic blood pressure and mean arterial pressure. In [6], the reliability of the pulse rate variability (PRV) module of a digital PPG-based telemedical system (SNC4ALL) was evaluated and the SNC4ALL PRV algorithm was compared to the well-known Kubios software [7]. The results obtained highlighted that PRV analysis performed on PPG pulse signals was in good agreement with ECG-based analysis in healthy subjects, both at rest and during the cold pressure test, and mainly when the features related to the investigation of low-frequency oscillations were computed. In diabetic patients, agreement was weaker than in healthy subjects, but good concordance was still obtained for some indices representing slow oscillations. The choice of the algorithm used for detecting the heartbeats within the PPG signal is of crucial importance in order to extract a PRV that is a reliable surrogate of the heart rate variability (HRV) obtained from the ECG signal. Bizzego et al. compared three methods: Derivative-Based Detection (DBD), Recursive Combinatorial Optimization (RCO), and Multi-Scale Peak and Trough Detection (MSPTD) [8]. The MSPTD algorithm [9] resulted in the most accurate algorithm among the three in discriminating heartbeats during high-magnitude body movements, such as cycling, but its computational complexity grew exponentially with the sampling frequency of the signal. Finally, all the investigated heartbeat detection algorithms appeared appropriate to process the cardiac component of the fNIRS data. Motion artifacts and data loss are two really frequent issues to be addressed during the pre-processing of PPG signals and derived parameters provided by wearables. Cajal et al. investigated data loss effects in HRV metrics using both simulated and real missing data [10]. The PPG-derived heart rate series were provided by Apple Watch during relaxing and stress-inducing experimental conditions. The proposed segment-based gap-filling method improved the reliability of the HRV features in case of scattered missing beats, especially for the frequency-domain metrics. Furthermore, the authors suggested discarding segments with more than 35% of missing beats or more than 20 s bursts to obtain errors lower than 20% in time-domain metrics and Poincaré plot parameters. In [11], Tsai et al. presented a systematic approach for the implementation of missing-feature imputation and ambiguous-feature resolution from the analysis of component waves of PPG signals acquired from the finger and the wrist. According to their findings, after the application of these techniques, the feature availability from PPG waveforms achieved more than 98.6%, with a significant correlation of up to 0.92 between finger and wrist PPG signals concerning the properties of the third and fifth component waves.

Among the growing plethora of wearable PPG sensor applications, clinical studies certainly play a leading role, considering the enormous recent advances in the field of telemedicine and e-health. In this Special Issue, the advantages of using the PPG signal were explored in several medical fields. PPG sensors integrated into wearable systems for light reflection rheography (LRR) could play a role in the early detection of deep vein thrombosis (DVT) in the lower limbs, as described in [12]. In this study, the occlusion of lower limb veins was simulated by pressuring a cuff up to 100 and 150 mmHg for slight and serious DVT scenarios, respectively. Under the serious DVT scenario, three parameters were able to classify positive or negative DVT states with an accuracy higher than or equal to 73%. A PPG signal can be also considered an additional biomarker for seizure detection. Glasstetter et al. explored the performance of wearable PPG-based identification of ictal tachycardia using the HR, finding good temporal agreement with the ECG-based method [13]. However, the authors highlighted a relevant negative effect of spontaneous movements on their findings when they attempted to identify ictal tachycardia in non-motor seizures. In clinical scenarios, when communicative functions are compromised, there is a strong need for reliable markers to assess pain levels in critically ill patients. In [14], the authors compared the results obtained through fourteen machine learning algorithms in terms of pain intensity classification, using PPG time, frequency and morphological features. An accuracy of 96.6% was reached in the discrimination of high pain and no pain levels using an artificial neural network on the data acquired from twenty-two healthy subjects during transcutaneous electrical nerve stimulation.

Given the growing use of wearable systems to monitor physiological signals during sleep, our Special Issue could not miss the contributions regarding this crucial aspect that significantly influences an individual’s quality of life. Lagazzera et al. presented UpNEA, a novel sleep-monitoring platform based on a smart glove, recording PPG, SpO2, and three accelerometer signals, a mobile application and a remote server [15]. The machine learning algorithms used for apnea and hypopnea detection showed promising results in highlighting sleep-disruptive breathing events and classifying them. For example, an accuracy value of 92.6% was found in the discrimination of central and obtrusive apnea. Castiglioni et al. investigated PPG and ECG signals acquired from twenty-one participants sleeping at high altitude (as a model of a sleep breathing disorder) and five alpine guides sleeping at sea level and extremely high altitudes [16]. The authors compared frequency features, multi-scale entropy and self-similarity of the PPG- and ECG-derived tachograms. Even if the finger-PPG systems resulted in measuring cardiovascular signals with sufficient quality in extremely high altitudes, their findings showed significant differences in the high-frequency power values and entropy metrics at short scales.

One of the most relevant advantages of wearable PPG systems is that thanks to their absolute non-invasiveness, they can be used during sports activities to monitor physical effort and prevent life-threatening situations. Although further testing is needed, an algorithm for the real-time processing of pulse oximeter and accelerometer data and recognition of pre-drowning symptoms in swimming pools was proposed in [17].

PPG signals are also currently used for biometric purposes, given the possibility of acquiring them easily and at low cost. A novel algorithm based on the study of PPG signal diffusive dynamics was reported in [18], and it was compared to eight existing techniques. The approach proposed reached the best equal error rate and processing times, considering 40 PPG signals measured with commercial devices.

As part of this Special Issue, new frontiers regarding PPG signal acquisition in contactless mode have been explored in the work of van Es et al. [19]. The authors compared eight algorithms for the extraction of PPG signals from face RGB videos, in terms of pulse rate and PRV features. The plane-orthogonal-to-skin (POS) and chrominance-based (CHROM) techniques were found to be the most robust for the assessment of autonomic dynamics by using remote-PPG, and Poincaré maps were suggested as the most reliable method to extract vagal dynamics information.

## 3. Conclusions

In conclusion, the contributions to this Special Issue allowed a varied journey into novel algorithms for PPG series processing and data analysis, new solutions for signal acquisition in both wearable and contactless modes, and interesting fields of application from clinical to sport and biometric monitoring.

All the findings reported reaffirm the need to continue refining existing techniques and proposing new approaches for the non-invasive acquisition and processing of PPG signals, which with its many strengths can continue to revolutionize our daily lives and clinical practices aimed at diagnosis and prevention.
